# Radiation-induced glioma following CyberKnife® treatment of metastatic renal cell carcinoma: a case report

**DOI:** 10.1186/1752-1947-6-271

**Published:** 2012-09-03

**Authors:** Malak Abedalthagafi, Ahmed Bakhshwin

**Affiliations:** 1 Pathology Department, Georgetown University Hospital, Washington, DC, 20007, USA

## Abstract

**Introduction:**

Post-stereotactic radiation-induced neoplasms, although relatively rare, have raised the question of benefit regarding CyberKnife® treatments versus the risk of a secondary malignancy. The incidence of such neoplasms arising in the nervous system is thought to be low, given the paucity of case reports regarding such secondary lesions.

**Case presentation:**

Here we describe a case of a 43-year-old Middle Eastern woman with primary clear cell renal cell carcinoma and a metastatic focus to the left brain parenchyma who presented with focal neurologic deficits. Following post-surgical stereotactic radiation in the region of the brain metastasis, the patient developed a secondary high-grade astrocytoma nearly 5 years after the initial treatment.

**Conclusion:**

Although the benefit of CyberKnife® radiotherapy treatments continues to outweigh the relatively low risk of a radiation-induced secondary malignancy, knowledge of such risks and a review of the literature are warranted.

## Introduction

Given the advent of new stereotactic radiosurgery techniques, important questions have arisen regarding the risk of secondary malignancy following such treatments. On the basis of previous case reports, the incidence of such a secondary malignancy following CyberKnife® therapy has been estimated at between 0.7% and 1.9% [1,2]. Specifically, reports of post- stereotactic radiation-induced central nervous system (CNS) tumors have been few, and reflect a higher incidence in patients with a predisposition to cancer, such as those with neurofibromatosis. Typically, such a secondary malignancy is thought to arise within a period of 5 to 10 years post-treatment, given a review of the literature involving such patients. Here we present a case of radiation-induced glioma in a patient following treatment with stereotactic radiosurgery for a metastatic renal cell carcinoma focus to the brain. It should be noted that although there remains a notable risk of developing a secondary CNS malignancy following radiotherapy treatment, it is thought that the overall benefits of such treatments outweigh the risk of developing a secondary neoplasm.

## Case presentation

**Figure 1:**
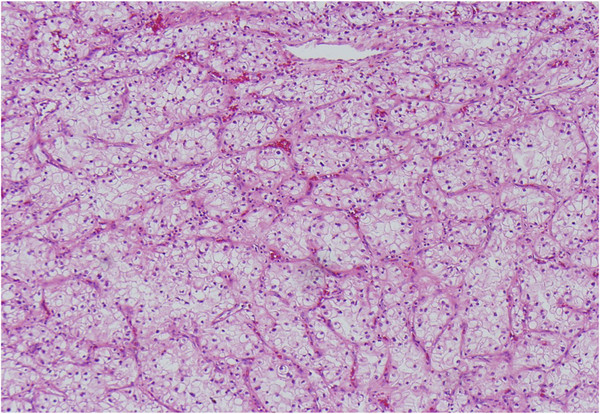
** Microscopic image of primary renal cell carcinoma.** Hematoxylin and eosin (H&E) stain at 20× magnification.

A 43-year-old Middle Eastern woman originally presented complaining of a sudden onset of right upper extremity weakness and numbness. A magnetic resonance imaging (MRI) scan of her brain demonstrated a lesion in the left frontal lobe consistent with a possible metastasis, and an abdominal computed tomography showed an 8cm mass in the right kidney. She underwent a radical laparoscopic nephrectomy a month later, and surgical pathology revealed clear cell renal cell carcinoma (Figure 1) of Fuhrman nuclear grade 3 without evidence of metastasis to perinephric fat, the adrenal gland, renal vasculature, or hilar lymph nodes.

**Figure 2:**
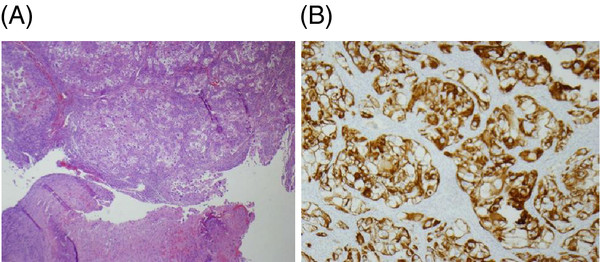
** Microscopic images of brain metastases.** (**A**) H&E stain at 10× magnification. (**B**) Keratin immunohistochemistry at 40× magnification.

Later that same month, the patient also had a left frontal craniotomy performed for metastatic focus resection. Microscopically, the specimen revealed nests of large pleomorphic cells with prominent eosinophilic nuclei that resembled malignant ganglion cells. However, clear cytoplasm was observed in some areas (Figure 2A). Immunohistochemical (IHC) staining was positive for keratin (Figure 2B), vimentin, and CD10, and negative for glial fibrillary acidic protein (GFAP), neurofilament, chromogranin, S100, Human Melanoma Black-45 (HMB-45), smooth muscle actin, desmin, and CD68. The brain mass was therefore diagnosed as poorly-differentiated grade 4 metastatic renal cell carcinoma. Following neurosurgery, the patient underwent whole brain radiotherapy followed by CyberKnife® stereotactic radiosurgery.

A year later, the patient began developing neurological symptoms including right arm tremor, weakness, dizziness, and abnormal sensation on her right cheek. An MRI scan of her brain revealed a large left frontal mass stemming from the insular that subsequently enlarged and grew upwards, creating a midline shift. After 5 months, she underwent resection of the mass. Pathology showed radiation necrosis with sheets of foamy macrophages and gliosis of the surrounding brain tissue with focal perivascular chronic inflammation. IHC staining was negative for keratin, with no evidence of viable tumor cells. IHC staining was positive for CD68 indicating the histiocytic nature of many of the foamy cells.

**Figure 3:**
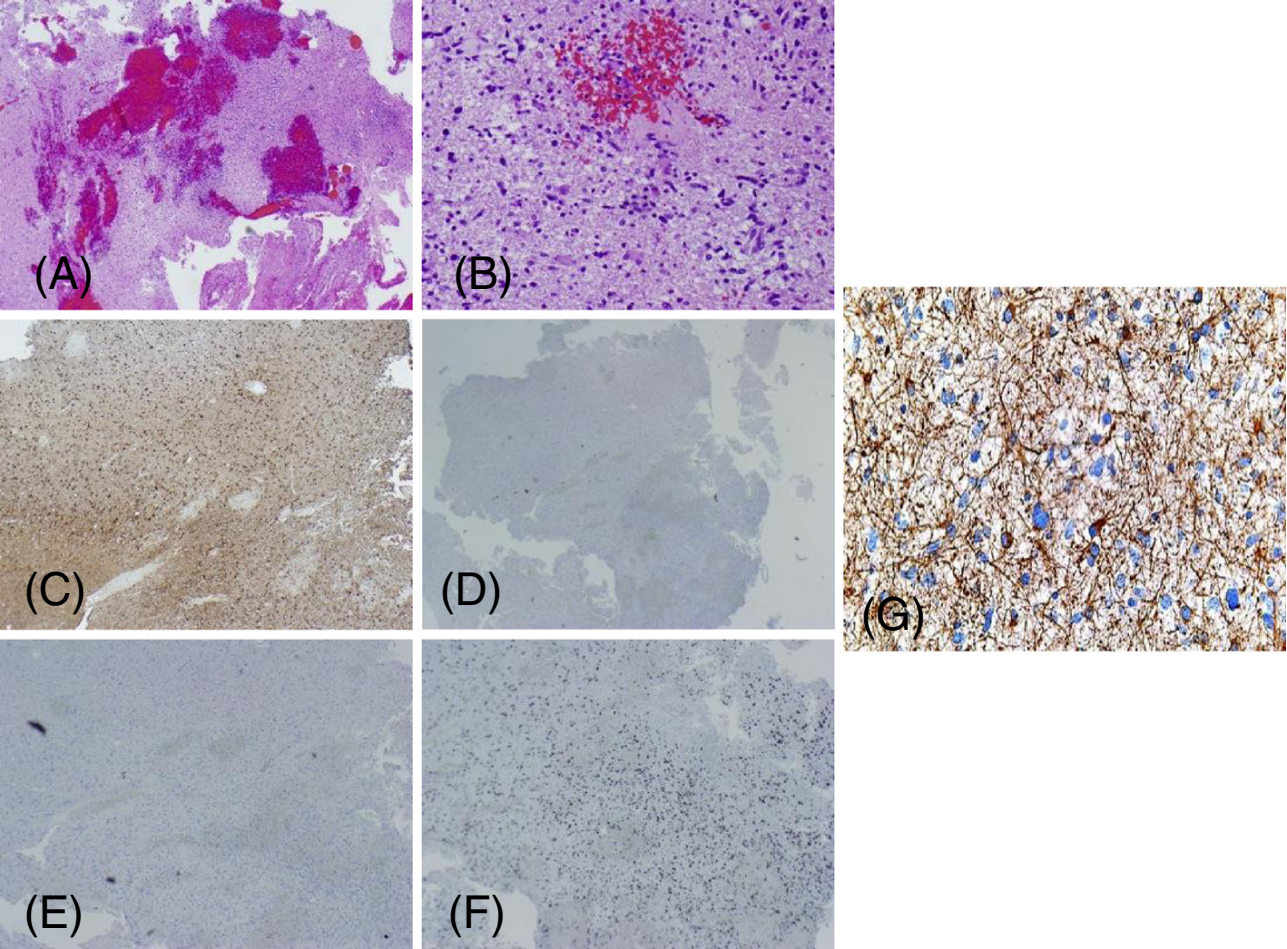
** Microscopic images of radiation-induced brain tumor.** (**A**) H&E stain at 10× magnification. (**B**) H&E stain at 20× magnification. (**C**) S100 immunohistochemistry at 10× magnification. (**D**) Keratin staining at 10× magnification. (**E**) CD10 staining at 10× magnification. (**F**) Ki-67 staining at 10× magnification. (**G**) GFAP staining at 10× magnification.

The patient once again presented with right-sided hemiparesis. Imaging studies showed a left occipital-parietal mass, and surgical resection was completed approximately 4.5 years after her initial craniotomy and brain irradiation. The pathologic appearance of the tissue was consistent with a high-grade astrocytoma, probably glioblastoma multiforme, with areas of necrosis and vascular proliferation (Figures 3A and 3B). IHC was also indicative of glial cells, with staining positive for GFAP and S100 (Figure 3C) but negative for keratin (Figure 3D) and CD10 (Figure 3E). The molecular immunology Borstel-1 (MIB-1) proliferation index was found to be 20% (Figure 3F).

## Discussion

The criteria for a radiation-induced neoplasm as originally outlined by Cahan *et al.* in 1948 [3] include: 1) the tumor must not be present at the time of irradiation; 2) there must be a prolonged latency period between radiation delivery and tumor development; 3) the tumor must arise in the irradiated region; 4) the tumor must be histologically distinct from the original tumor; and 5) the patient must not have a genetic predisposition to the development of cancer. Our patient’s case seems to fulfill all of these criteria. Extensive imaging was performed at the time of the original diagnosis and no evidence of a lesion was found in the left occipital-parietal area. Although 5 years is the generally accepted minimum latency period for developing a radiation-induced malignancy, cases with shorter latency periods have been reported. This case is complicated by the fact that the patient received both whole brain irradiation as well as Gamma Knife stereosurgery, but the affected secondary location was definitely within the irradiated field. Tumor markers show the histologic disparity between the original brain metastases and the new lesion. Finally, the patient was not known to have any genetic conditions that predispose toward carcinogenesis.

The risk of developing a secondary nervous system cancer, particularly meningiomas, following conventional fractionated radiation exposure has been well established. Studies of the survivors of the atomic bombings of Hiroshima and Nagasaki indicate an increased incidence of meningiomas in this population [4-6]. Epidemiological data derived from child immigrants to Israel after World War II who received radiation for the treatment of tinea capitis showed an increase of up to 6.9-fold in nervous system neoplasms (including meningiomas, gliomas, and nerve sheath tumors) [7]. There has also been some suggestion of greater numbers of meningiomas and gliomas in adults who underwent radiotherapy for pituitary adenomas [8,9]. Finally, experiments on primates given therapeutic doses of fractionated whole-brain radiation resulted in high rates of induction of glioblastoma multiforme [10], and over 100 human cases in which a glioma appeared after radiotherapy have been identified [11]. Nevertheless, the absolute risk of developing a radiation-induced neoplasm after receiving radiotherapy to the CNS remains relatively low and it is generally thought that the overall benefits of the treatment outweigh the negative complication rate of alternative treatments [12].

The risk of oncogenesis due to stereotactic radiosurgery has generally been believed to be lower than that of conventional radiotherapy. Although traditional practices involve low-dose radiation delivered to a high volume of tissue, stereotactic methods allow for high-dose, low-volume radiation with a steep drop in dosage outside the targeted zone. The perception of increased safety in radiosurgery has been supported by an analysis of nearly 5000 English patients who underwent Gamma Knife therapy [13]. This study found only one new case of astrocytoma following radiation in comparison to a predicted incidence of 2.47 in the general population. A major criticism of the study has been that the mean follow-up interval was only just over 6 years. However, the follow-up of over 1200 of the patients was greater than 10 years.

## Conclusions

As yet, relatively few case reports of post-stereotactic radiation-induced nervous system tumors have been documented in the literature (Table 1), and some of these involve patients with a genetic predisposition to cancer (e.g. neurofibromatosis 2) who thus do not completely fulfill the Cahan [3] criteria. On the basis of these reports, the incidence of a secondary malignancy following stereotactic radiosurgery has been estimated at between 0.7% and 1.9% [1,2]. In addition, it is believed that, due to the relatively recent advent and dissemination of this technology, relatively more cases may be identified in the near future as a greater population of treated patients reaches the most dangerous latency interval.

**Table 1:** Case reports of secondary malignancy following stereotactic radiosurgery

**Reference**	**Patient**^**a**^	**Indication for radiation**	**Location of primary lesion**	**Latency period**	**Secondary malignancy**	**Location of secondary lesion**
Comey 1998 [14]	44M	VS	Cerebellopontine angle	5 years	Triton tumor	Cerebellopontine angle
Noren 1998 [15]	18F^b^	VS	Cerebellopontine angle	6 years	Triton tumor	Cerebellopontine angle
Thomsen 2000 [16]	19F^b^	VS	Cerebellopontine angle	6 years	Meningosarcoma	Cerebellopontine angle
Yu 2000 [17]	70F	Meningioma	Occipital region	7 years	GBM	Occipital lobe
Kaido 2001 [18]	20M	AVM	Right parietal lobe	6.5 years	GBM	Right parietal lobe
Shamisa 2001 [19]	57F	VS	Cerebellopontine angle	7.5 years	GBM	Inferior temporal lobe
Bari 2002 [20]	30F^b^	VS	Cerebellopontine angle	3.5 years	Malignant nerve sheath tumor	Cerebellopontine angle
Salvati 2003 [11]	66F	Cavernoma	Right frontal region	13 years	GBM	Right frontal, corpus callosum
Shin 2002 [21]	26F	VS	Cerebellopontine angle	6 years	Malignant nerve sheath	Cerebellopontine angle
McIver 2004 [22]	43F	Metastatic melanoma	Left parietal parasagittal region	5.3 years	Anaplastic astrocytoma	Cerebellum
Sanno 2004 [23]	53F	Meningioma	Falx cerebri	5 years	Osteosarcoma	Right parietal lobe
Sheehan 2006 [1]	7M	AVM	Right basal ganglia	12 years	Assumed meningioma (?)	Right lateral middle cranial fossa
Sheehan 2006 [1]	16F	AVM	Right temporal	10 years	Assumed meningioma (?)	Right temporal lobe
Balasubramaniam 2007 [24]	64F^b^	AVM	Cerebellopontine angle	5 years	GBM	Right temporal lobe
Berman 2007 [25]	34F	AVM	Pineal region	9 years	GBM	Corpus callosum and/or right parietal lobe
Carlson 2009 [26]	15F^b^	AVM	Bilateral posterior fossae	10 years	Rhabdomyosarcoma	Inferior right medulla and cerebellomedullary cistern

^a^age at time of radiation.

^b^patient with neurofibromatosis 2.

Abbreviations: *AVM* arteriovenous malformation, *F* female, *GBM* glioblastoma multiforme, *M* male, *VS* vestibular schwannoma.

## Consent

Written informed consent was obtained from the patient for publication of this case report and accompanying images. A copy of the written consent is available for review by the Editor-in-Chief of this journal.

## Competing interests

The authors declare that they have no competing interests.

## Authors’ contribution

MA analyzed and interpreted the patient data regarding the histological and immunohistochemical tests, and was a major contributor in writing the manuscript. AB helped in analyzing patient data and follow-up history. Both authors read and approved the final manuscript.
